# Climate change can disproportionately reduce habitats of stream fishes with restricted ranges in southern South America

**DOI:** 10.1038/s41598-024-66374-6

**Published:** 2024-07-09

**Authors:** Gustavo Bizama, Arif Jan, J. Andrés Olivos, Guillermo Fuentes-Jaque, Claudio Valdovinos, Roberto Urrutia, Ivan Arismendi

**Affiliations:** 1 Doctorado de Ciencias Ambientales, en Ecosistemas Acuáticos Continentales, Facultad de Ciencias Ambientales, Centro EULA-Chile, Universidad de Concepción, Víctor Lamas 1290, 4070386 Concepción, Chile; 2 Centro de Recursos Hídricos para la Agricultura y Minería CRIHAM, Concepción, Chile; 3 Department of Fisheries, Wildlife, and Conservation Sciences, Oregon State University, Corvallis, OR 97331 USA; 4 Department of Environmental Sciences and Renewable Natural Resources, Faculty of Agricultural Sciences, University of Chile, Santiago, Chile

**Keywords:** Ecology, Zoology, Climate sciences, Environmental sciences, Limnology

## Abstract

Freshwater fishes are among the most threatened taxa worldwide owing to changes in land use, species introductions, and climate change. Although more than half of the freshwater fishes in the Chilean Mediterranean ecoregion are considered vulnerable or endangered, still little is known about their biogeography. Fishes of the family Perciliidae are endemic of this region and ideal cases to study potential implications of global warming given their endangered conservation status, small size, restricted range, and limited dispersal capacity in fragmented habitats. Here, we model the spatial distribution of habitats for *Percilia irwini* and *P. gillissi* under current (1970–2000) and future (2050–2080) climatic scenarios (SSP245, SSP585). We implement maximum entropy (MaxEnt) models adapted for stream networks using high-resolution datasets of selected geophysical and climatic variables. At present, both species inhabit relatively low-quality habitats. In the future (SSP585), suitable habitats for *P. irwini* are predicted to be reduced drastically (99%) with potential local extirpations in its northern range. Similarly, up to 62% of suitable habitats for *P. gillissi* would also be reduced in the future. Our study provides insights about assessing future threats and vulnerability of endemic, endangered, range-restricted, and small-bodied freshwater species in this region and elsewhere.

## Introduction

Freshwater fauna are among the most threatened taxa worldwide owing to human-related disturbances including changes in land use, introduction of exotic species, and climate change^1–3^. In the Southern Hemisphere, studies on the impacts of climate change in freshwaters are scarce despite many species having serious conservation issues^2,3^. Indeed, the potential contraction and fragmentation of habitats are important aspects to consider for understanding the resilience of sensitive taxa to environmental change^4,5^. For example, species with restricted ranges, often associated with cold climates, are highly vulnerable to climate change^6,7^. In contrast, taxa with extended ranges might be less vulnerable and potentially more resilient to global warming^8^. In this context, understanding the potential impacts of climate change on species distribution is crucial for effective conservation planning^9^. In data-deficient regions of the world, predictive spatial-statistical models can serve as cost-efficient tools to inform the conservation and management of freshwater biodiversity under scenarios of environmental change^10,11^.

Species distribution models (SDMs) are spatially explicit representations of species distributions based on statistical relationships between species occurrences and environmental variables^12,13^. This approach is particularly useful for understudied species occupying data-poor regions^11,12,14^. SDMs can be used as the first step to develop hypothesis-based research and projections to better understand changes in species distributions under future global environmental scenarios^15,16^. These models can serve as the first approach to understand the responses of relatively vulnerable ecosystems and organisms to climate change as the case of endemic fishes from Mediterranean regions^17–19^.

The Chilean Mediterranean ecoregion (27°–36° S) has been described as susceptible to be adversely affected by climate change^17,20,21^. Recent long-term megadroughts^22^ have affected higher elevation areas^23^ threatening the biodiversity of freshwaters in the region^18,24^. This is especially important considering that more than 50% of freshwater fishes are classified as endangered^25^. Native fishes from the Chilean Mediterranean ecoregion have relatively small (< 20 cm total length) body sizes^26,27^ and limited dispersal capacity (< 30 km)^28^ with many endemic species having restricted ranges and low population abundances^27,29,30^. Among them are the two endemic ‘Carmelitas’ including *Percilia irwini* (Eigenmann, 1928) and *P. gillissi* (Girard, 1855)^25^. *P. irwini* is a benthopelagic endemic species to the Bío-Bío River Basin (36°35′ S–38° 44′ S) inhabiting mostly relatively shallow (˂ 1.0 m) pool habitats^29,31^. *P. gillissi* have a broad distribution between Estero Limache and Puerto Montt (32° 55′ S–41° 28′ S). This species is pelagic and inhabit riverine environments associated to both cascade and shallow pool habitats^27,30,32^. These two ‘Carmelitas’ have a small body size, up to 10 cm total length^26,29^; their dispersal is limited especially in fragmented habitats^33^. ‘Carmelitas’ represent ideal study cases to document the effect of climate change on endemic, endangered, range-limited, and small-bodied freshwater species.

Here, we model current and future distribution of habitats for these two ‘Carmelitas’ using SDMs under two different scenarios of global warming. We hypothesized that these species would have contractions in their distribution mostly in their northern portion of their ranges and lower elevation areas. This is due to the climate is naturally warmer and more arid in the north compared to the south, and global warming^18,34^ would further exacerbate climatic differences between northern and southern areas. In addition, the low dispersal capacity of these species^28,33^, and their current distributions in already human-disturbed areas located at lower elevations^24,27,35^ will negatively affect existing habitats. Our work illustrates the use of SDMs and publicly available datasets to assess the potential consequences of global warming on native endemic freshwater species with restricted ranges.

## Results

The resulting SDMs for *P*. *irwini* and *P. gillissi* showed a relatively good performance with the Area Under the Curve (AUC) values of 0.86 and 0.83 respectively. Of the nine variables selected for the best-supported models (Supplementary Table S1); Strahler order, Mean Temperature of Coldest Quarter (bio11), and Average Annual Precipitation (bio12 hydro) at the basin level had the greatest contribution towards model fit (Supplementary Table S1).

### Modeled present and future distribution of habitats for *P. irwini* and *P. gillissi*

**Figure 1:**
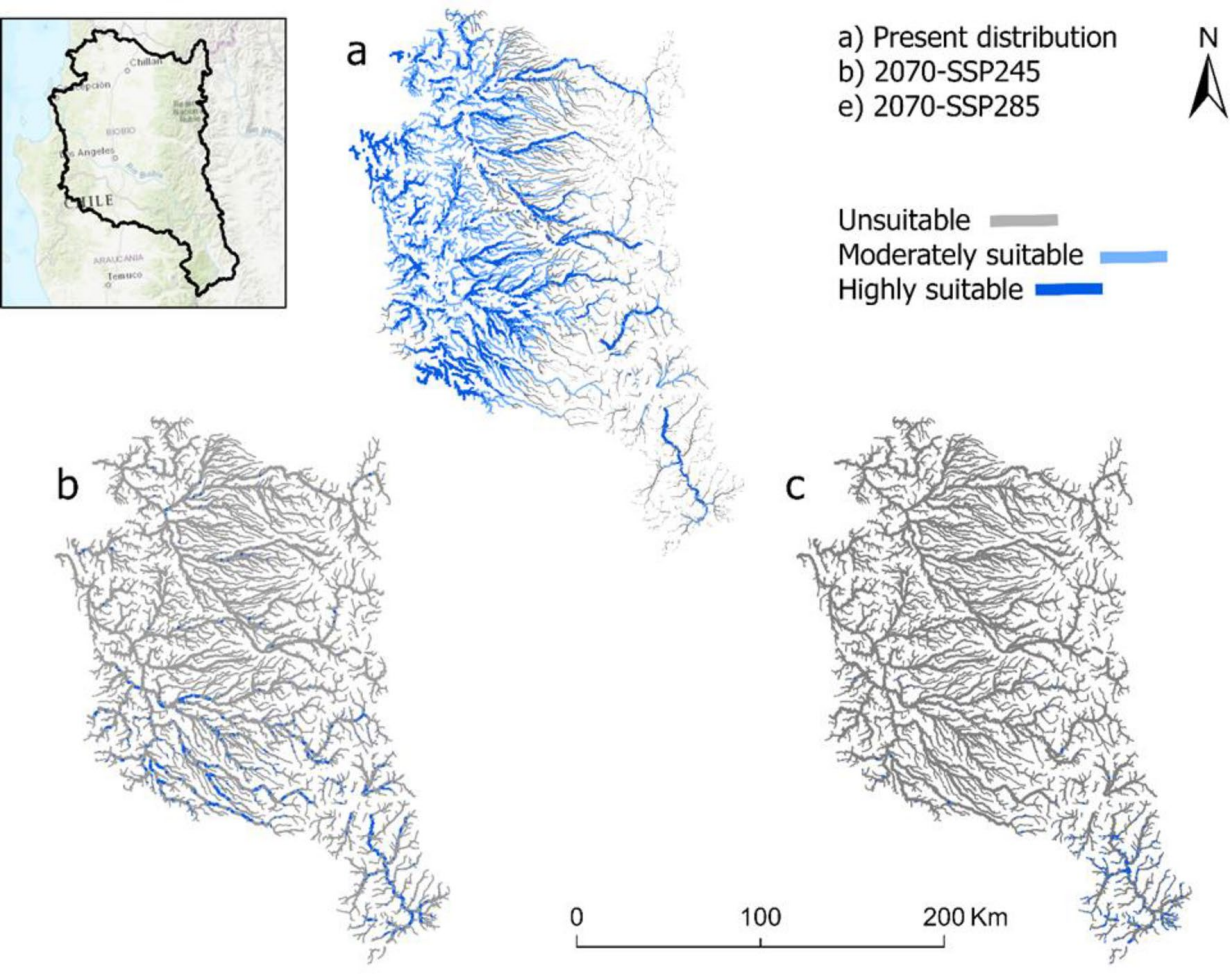
Distribution of habitats for *P. irwini* based on the best-supported MaxEnt model. (**a**) Present (**b**) 2070 for an intermediate scenario of climate change (SSP-245), and (**c**) 2070 for an extreme scenario of climate change (SSP-585). For the *P. irwini* model, a minimum training presence threshold and defined as unsuitability was *P* ≤ 0.06. Moderately suitable habitats 0.06 *P* ≤ 0.5 and highly suitable habitats were classified as *P* > 0.5. The figure was produced with ArcGIS Pro 3.0.0 with extensions provided by Oregon State University (https://www.esri.com/en-us/arcgis/products/arcgis-pro/overview).

Model outputs of present distribution including moderately and highly suitable habitats for *P. irwini* were mostly restricted around low to middle elevation areas (Fig. 1a). In 2070, both scenarios (SSP245 and SSSP585) of climate are predicted to substantially decrease the amount of suitable habitats for *P. irwini* compared to the present (20,200 km). This resulted in a reduction of 19,105 km and 19,785 km of suitable habitats for the intermediate and extreme scenarios, respectively (Fig. 1b,c). In the future, moderately and highly suitable habitats would be restricted mainly to the south (Bio-Bío River basin) with highly fragmented sections scattered around the range of *P. irwini.*

**Figure 2:**
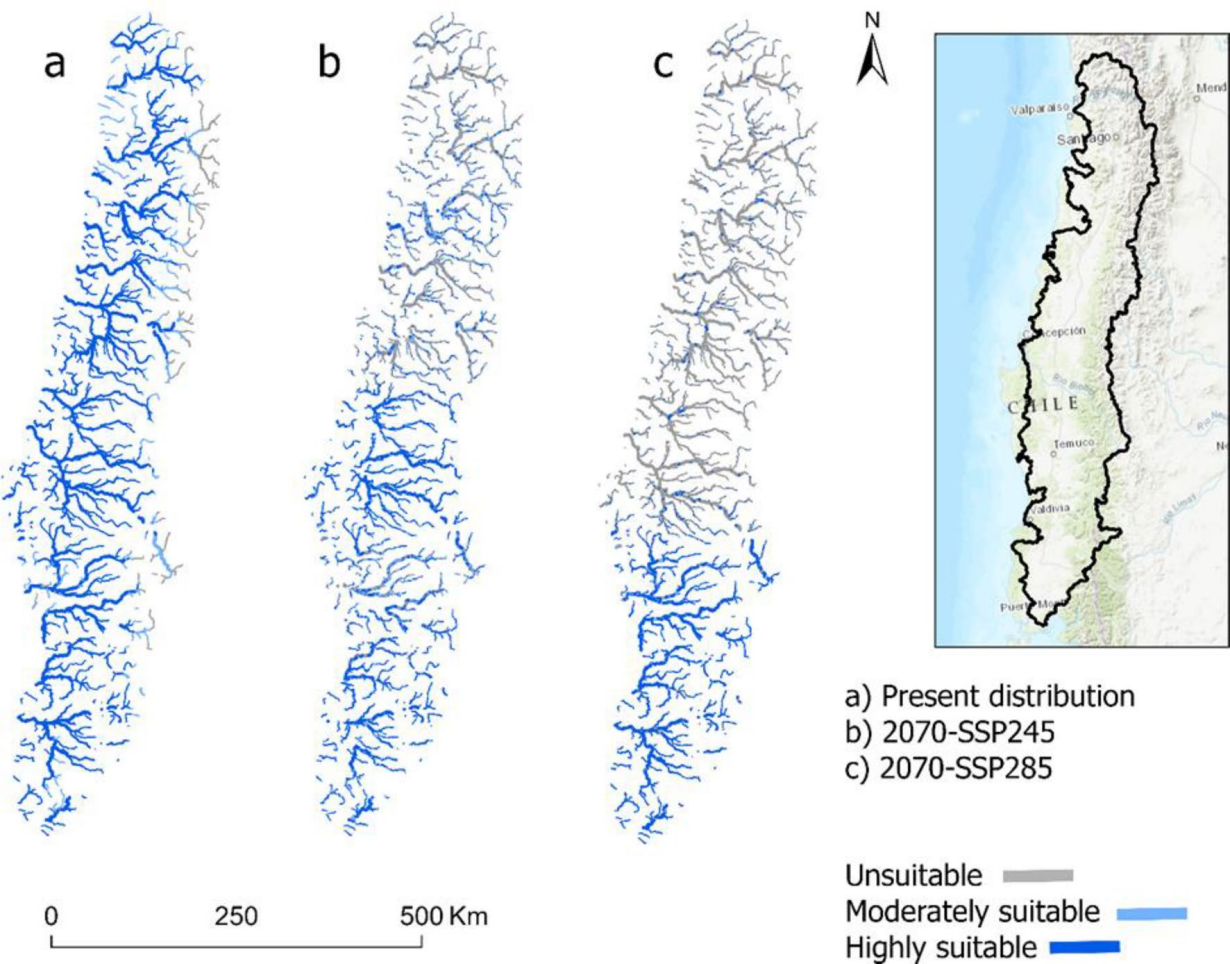
Distribution of habitats for *P. gillissi* based on the final MaxEnt model. (**a**) Present (**b**) 2070 for an intermediate scenario of climate change (SSP-245), and (**c**) 2070 for an extreme scenario of climate change (SSP585). For the *P. gillissi* model, a minimum training presence threshold was defined as unsuitability *P* ≤ 0.04. Moderately suitable habitats 0.04 *P* ≤ 0.5 and highly suitable habitats were classified as *P* > 0.5. The figure was produced with ArcGIS Pro 3.0.0 with extensions provided by Oregon State University.

The distribution of both moderately and highly suitable habitats for *P. gillissi* at present (123,348 km) was relatively extended within the range of this species (Fig. 2a). In contrast, the distribution of potential habitats in the future under an intermediate scenario of climate change included the loss of 42,560 km of suitable habitats in the northern portion of the range of *P. gillissi* (Fig. 2b). Similarly, the predicted distribution of habitats in the future under an extreme scenario of climate change included the loss of 72,082 km of suitable habitats (Fig. 2c). Under this scenario, moderately and highly suitable habitats were located mostly in the south (Bio-Bío, Imperial, Toltén and Valdivia rivers basins) with highly fragmented sections dispersed within the range of *P. gillissi*.

### Modeled densities of present and future habitats for *P. irwini* and *P. gillissi*

**Figure 3:**
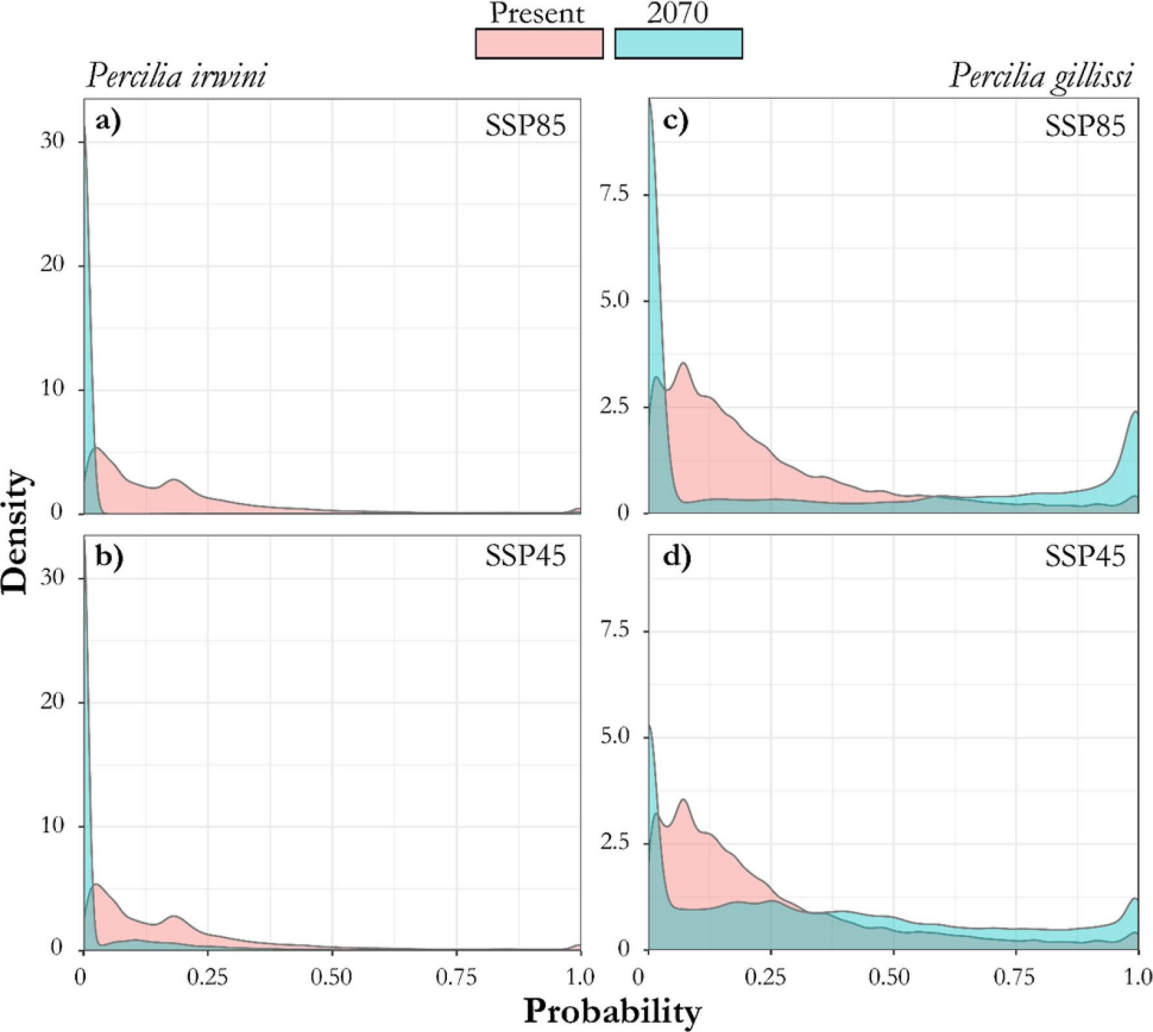
Density of habitats by their probabilities for *P. irwini* (**a**, **b**), and *P. gillissi* (**c**, **d**) under present and future scenarios of climate change. The X axis corresponds to the range of probabilities of the SDM (stream reach = 15 m pixels), and the Y axis represents the number of pixels with probability values in the range of each species. Climatic change scenarios include one extreme (SSP585) and one intermediate (SSP245) scenario.

The present density of habitats for *P. irwini* (Fig. 3a,b) illustrated a relatively low availability of highly suitable environments (*P* > 0.5). Rather, most habitats were moderately suitable (0.06 < *P* ≤ 0.5). Under future scenarios of climate change, the density of unsuitable habitats for this species will increase. For *P. gillissi* (Fig. 3c,d), the present density of habitats was more broadly distributed compared to *P. irwini*. Under future scenarios of climate change, the density of unsuitable habitats for this species increased, but this was also the case for highly suitable habitats.

### Shifts in elevation of suitable habitats for *P. irwini* and *P. gillissi* under future climate change scenarios

**Figure 4:**
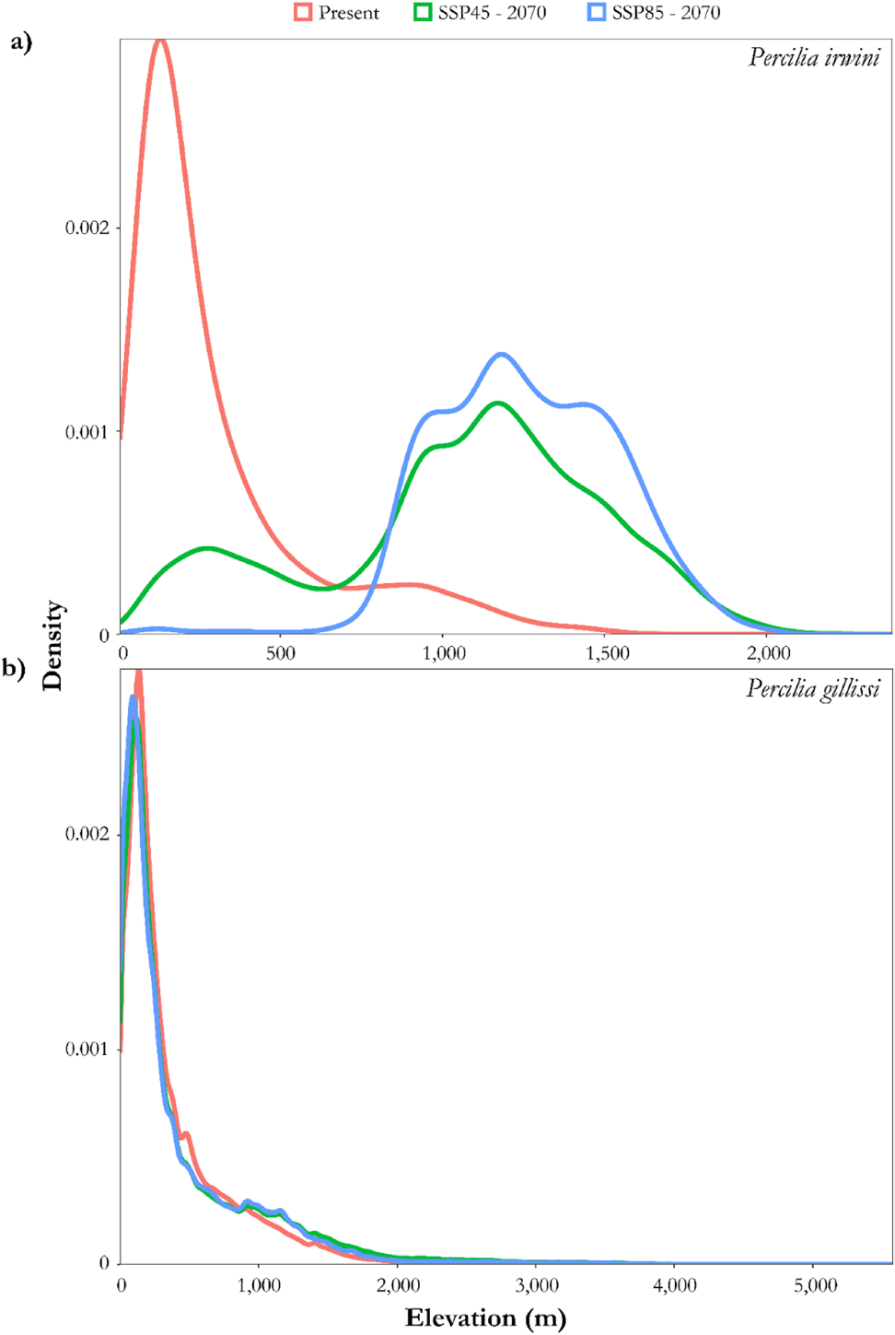
Density plots of suitable habitats by elevation for *P. irwini* (**a**) and *P. gillissi* (**b**). The X axis corresponds to the elevation range of the stream reaches (15 m pixels) and the Y axis represents a density of pixels in the range of each species. Climatic scenarios include one extreme (SSP585) and one intermediate (SSP245) climate change scenario.

The density of suitable habitats (*P* > 0.06) by elevation for *P. irwini* (Fig. 4a) between periods consistently shifted toward higher elevation areas (800–2000 m.a.s.l.) by 2070 under the two climate change scenarios. In the case of *P. gillissi* (Fig. 4b), suitable habitats (*P* > 0.04), appeared to be relatively similar below 2000 m.a.s.l. between both periods and scenarios of climate change.

### Habitat gains and losses under future climate scenarios for *P. irwini* and *P. gillissi*

**Figure 5:**
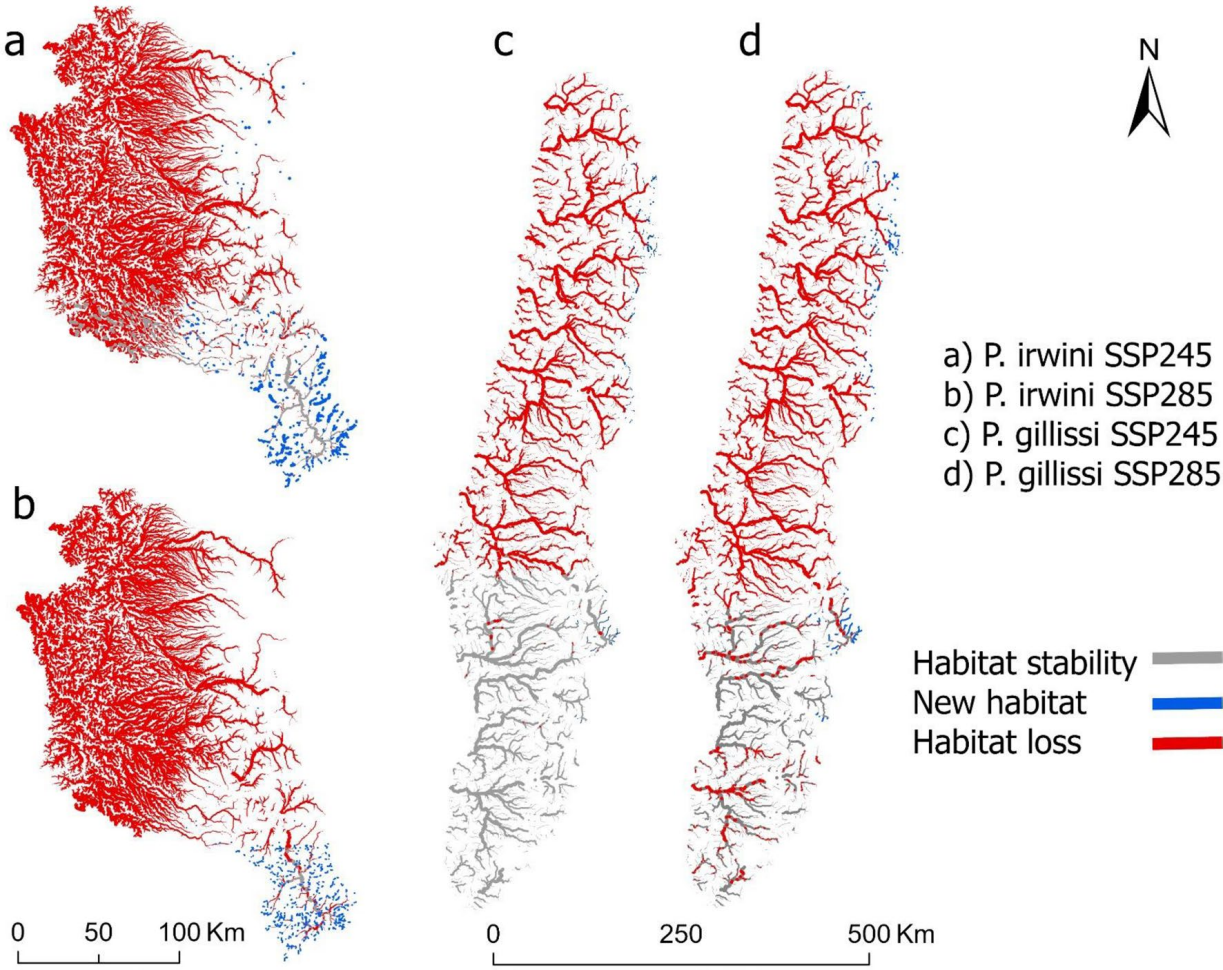
Distribution of moderately and highly suitable habitats between periods (present vs. 2070) including habitats (no change between present and 2070), habitat new (not suitable in present and suitable in 2070), and habitat loss (suitable in present and not suitable in 2070) for *P. irwini* (**a**, **b**) and *P. gillissi* (**c**, **d**). Includes two climatic scenarios for 2070 (one extreme SSP285 and one intermediate SSP245). For this intersection, the threshold cutoff of 0.06 for *P. irwini* and 0.04 for *P. gillissi* was used. The figure was produced with ArcGIS Pro 3.0.0 with extensions provided by Oregon State University.

The changes in the distribution of moderately and highly suitable habitats between the present and 2070 based on two scenarios of climate change for *P. irwini* (Fig. 5) showed loses between 92 and 99% under the moderate and extreme climate change scenarios, respectively (Fig. 5a,b). Only, a small percentage of habitats was not predicted to change under the two scenarios of climatic change (2–8%) and additional habitats (0.6–12%) were available at higher elevation areas in the southern portion of the map (Fig. 5a,b). For *P. gillissi*, the loss of habitat in both scenarios is predicted to be between 42 and 62% under the moderate and extreme climate change scenarios, respectively (Fig. 5c,d). Suitable habitats that are predicted to stay relatively stable fluctuated between 38 and 58%, whereas new habitats (5.5–7.8%) occurred in higher elevation areas at the east side of the region under the two climate change scenarios, respectively.

## Discussion

We use species distribution models to map present and future suitable habitats of two endemic freshwater fishes with restricted ranges in Chile, including *P. irwini and P. gillissi*. Both species are currently classified as endangered with decreasing population trends^36,37^. Our findings indicate that both species are currently using moderately suitable habitats, but future projections of climatic change might lead to remarkable declines in the availability of suitable habitats. Future habitat changes are more detrimental for *P. irwini* than for *P. gillissi*, and only a small fraction south of the Bio-Bío River is expected to maintain suitable conditions for *P. irwini*.

The current distribution of suitable habitats for *P. irwini* and *P. gillissi* relies mostly on a relatively moderate habitat quality. It is possibly these species have been exposed to changes in climate in the past, even from recent decades^38,39^, but is still unclear they would be able to persist in the future under more extreme climatic changes^40^. Similar issues have been reported elsewhere^41,42^. The lack of basic knowledge about the physiology and thermal tolerances of these endemic species makes it difficult to forecast their vulnerability without a high degree of uncertainty. The availability of suitable habitats under both future (2070) climatic scenarios indicates considerably high contractions of climatic niches with 92–99% for *P. irwini* and 42–62% for *P. gillissi*. These climatic scenarios would lead to drastic habitat losses in the northern portion of the ranges, where higher rates of climate warming are expected^23,43^ *P. irwini* and *P. gillissi* are congeneric allopatric species with different habitat requirements. *P. irwini* are adapted to small, shallow, and slow waters with a preference for pools habitats^30,31^. Potential climatic refuges located in headwaters at elevated areas might be key for this species to persist in the future. Conversely, *P. gillissi* are highly plastic^32^ and thus, its potential broader niche would allow this species to persist under more extreme climatic scenarios without having the need to migrate to higher elevation areas.

Significant habitat contractions are expected to occur in the northern portion of the range of *P. gillissi* suggesting potential local extirpations in some basins from the Aconcagua River to the Bio-Bío River. Currently, population declines in both species have occurred due to other human-related impacts such as pollution from industrial and domestic effluents^30,31^. Previous work^30,44^ have suggested that *P. irwini* and *P. gillissi* are very sensitive to other human-related impacts such as invasive species^24,45^ and habitat fragmentation by hydroelectric dams^33,46,47^,. These activities could act synergistically with climate change^48^. Habitat fragmentation can limit the ability of species to track their preferred climatic conditions and reduce the availability of suitable habitats^49^. Invasive species can compete with native species for resources and predation, which can further reduce the abundance and distribution of native species^50^. Therefore, managing these other stressors is crucial for the conservation of biodiversity in the face of climate change^51^. Collectively, these multiple stressors have the potential to accelerate the contraction of habitats and further exacerbate the vulnerability of these species to global environmental change.

Suitable habitats for *P. irwini* and *P. gillissi* are expected to remain or slightly increase in the future only in higher elevation areas. These areas represent potential climatic refuges, but they seem insufficient and highly isolated due to their low connectivity, especially in the case of *P. irwini.* As seen in other cases, migrations toward colder and higher elevational areas are potential consequences of global warming^3,52,53^. Heggenes et al*.*^54^ and Kelley et al*.*^55^ point out how fundamental these migrations are in river systems, underpinning adaptive capacity to disturbances, as well as functional connectivity within them. Unfortunately, human-related impacts in the region are concentrated in lower elevational areas^56^. Therefore, suitable habitats in higher elevational areas seem the only option for climatic refuges^56,57^. However, the ability of species to track their preferred conditions may be limited by various factors, including their dispersal ability, the availability of suitable habitats, and the presence of barriers to movement^49^. Some species may not be able to keep pace with the rate of climate change and may experience range contractions or even extinction^58^. This is particularly a concern for species with narrow climatic niches and limited dispersal abilities, such as many freshwater fish species^3^ including *P. irwinii* and *P. gillissi*.

In our study area, the topography of rivers and their climate seem highly influential to define suitable habitats for *P. irwini* and *P. gillissi*. The importance of these variables has been documented for other cases when using high-resolution spatially explicit models^59^. Both the geophysical template and climate are fundamental to the biogeographic characteristics of the isolation and resulting endemism of the Chilean ichthyofauna^27^. Keppel et al*.*^60^ suggest that areas of high biodiversity and endemism with relict species could be refuges in the past and buffers to future climate changes. However, the expected increases in extreme temperature conditions, changes in precipitation patterns, and a decrease in the availability and connectivity of habitats might displace buffer areas toward higher elevations^61^. Unfortunately, the higher increases in temperature in the highest areas of the Chilean Mediterranean region^23^ might limit these potential new climatic refuges. The latter, in conjunction with human impacts and temperature increases in lower elevational areas, could create an ecological trap for the fish fauna of central Chile.

## Limitations

Our modelling approach has some limitations. For example, biases when only public biodiversity databases are used (e.g., spatial congruence among occurrences)^62^ might affect model performance. We used multiple datasets including GBIF in conjunction with additional sources from the Chilean Government. This combination of sources resulted in relatively good performance of best supported MaxEnt models (AUC > 0.8). In addition, as indicated by Furby and Araújo^63^, the lack of consideration of the effects of land uses in SDMs could over-or-underestimate the tolerance capacity of species, affecting the capacity of models to be used across multiple time periods. However, our objective is to provide preliminary models, which also included land cover categories as covariates. We document biases towards the urban (i.e., more accessible sites for sampling) and water (i.e., lakes and reservoirs) classes and given these potential issues, we do not include land cover categories in the best supported MaxEnt model. Further, we include 88 variables that represent a suite of characteristics including topography, hydrology, bioclimatic, and land use changes. The high-resolution layers (15 m) we use likely provide a better representation of both environments for species with restricted distributions and greater detection of climate variability. Our selected modeling approach likely does not overestimate habitat extirpation as a common problem reported when low-resolution layers are used^64,65^. Furthermore, future research can use similar approaches focusing on other areas where most of the species are considered endangered^25^. Moreover, mapping potential climatic refuges can assist the potential translocation of species as well as the development of field surveys to test and validate our findings (e.g., monitor the abundance of populations in moderately and highly suitable habitats).

Lastly, more research is needed to conduct experiments of thermal tolerance of these and other endemic species.

## Conclusions

In conclusion, our study reveals the potential severe impacts of climate change on the distribution of endangered fish species, *P. irwini* and *P. gillissi*, in Chile's freshwater ecosystems. Both species face significant habitat loss and fragmentation under future climate scenarios, suggesting future local extirpation of *P. irwini*. Higher elevation areas could serve as climatic refuges, but their limited availability and connectivity pose challenges. Human impacts, such as habitat fragmentation and invasive species, exacerbate these species' vulnerability when combined with climate change. Our findings underscore the urgent need for climate-informed conservation strategies. Future research should focus on the potential impacts of climate change on other freshwater species in Chile and the effectiveness of different conservation strategies. Detailed studies on potential climatic refuges and their connectivity could inform conservation strategies. Proactive management is needed to ensure the persistence of *P. irwini* and *P. gillissi* in a rapidly changing climate.

## Materials and methods

### Study area

**Figure 6:**
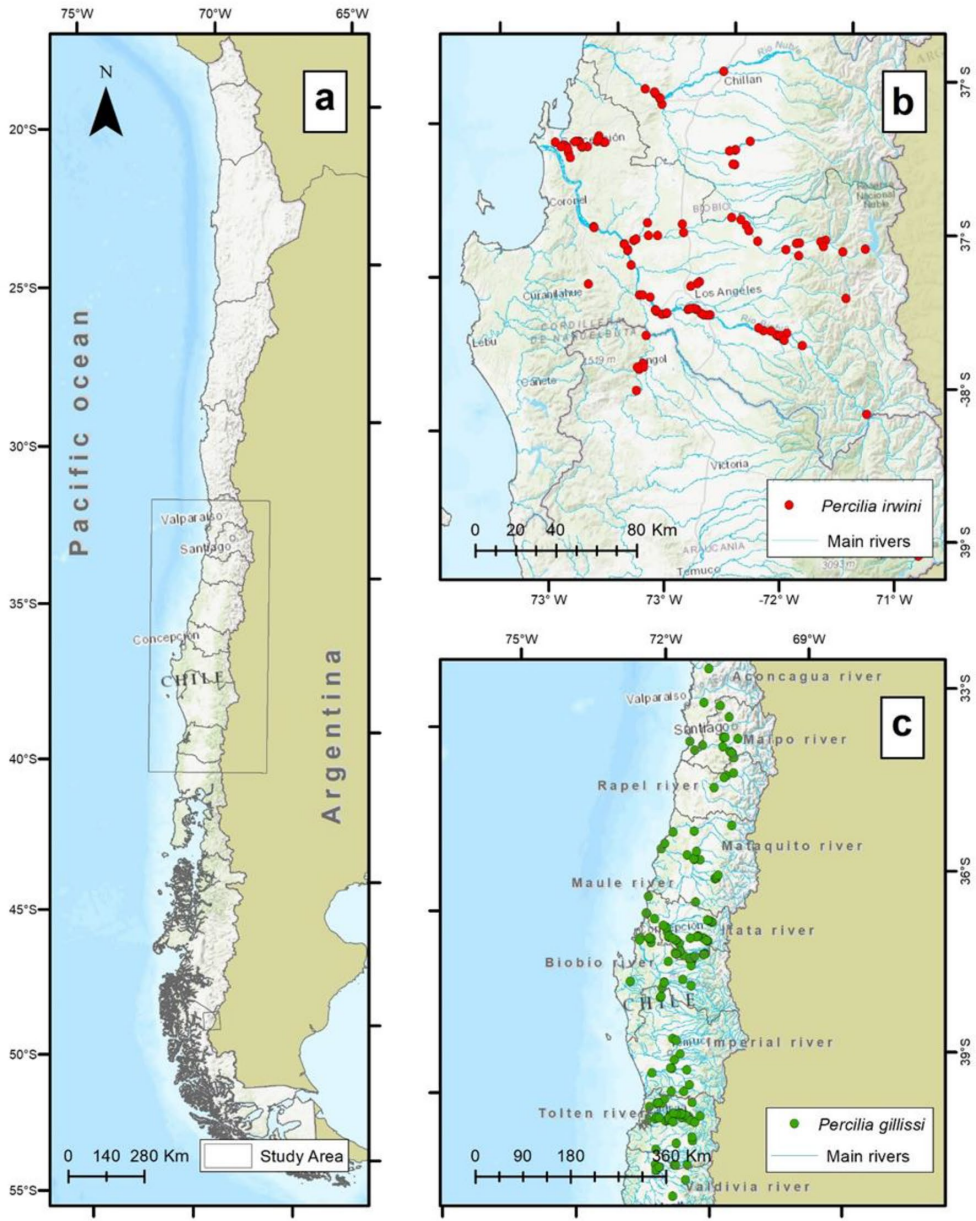
The study area in the south-central zone of Chile (**a**). Distribution of occurrences for *P. irwini* (**b**) and *P. gillissi* (**c**). The figure was produced with ArcGIS Pro 3.0.0 with extensions provided by Oregon State University.

We conducted our study in Chile (Fig. 6a) and included two study areas based on local records that described the distribution of the two endemic species *P. irwini* and *P. gillissi*. Our study region, supports a significant fraction of Chile's population (80%) and agricultural activities (85%) and therefore, play a key role in the country's socioeconomic activities^17,20,66^.

The range of *P. irwini* is limited to the VIII Region of Biobío in the center-south zone of Chile (Fig. 6b). This study area extended from the Itata River Basin in the north (35° and 37° S) to the Biobío River Basin (37° and 39° S) in the south, with a total area of ~ 35,294 km^2^. Both basins are influenced by a precipitation regime of a mixture between snow and rain^66^, resulting in a Mediterranean climate dominated by vegetation formations of evergreen forest in higher elevation areas and sclerophyllous scrub in lower elevation areas^67^. These basins have been affected by the replacement of native forests by plantations of exotic species and an increase in agriculture, with a concentration of both rural and urban areas in the lower portions of the basins^20,66^.

*P. gillissi* have a broader distribution from 32° to 41° S, and includes the Andean basins of the Aconcagua, Maipo, Rapel, Mataquito, Maule, Itata, Biobío, Imperial, Toltén and Valdivia rivers, extending to the south of the Mediterranean area of central Chile (Fig. 6c). Each of these basins is not connected to each other and drains directly into the Pacific Ocean. The flow regimes are a mixture of rain and snowmelt with fast water velocities due to the steep slopes in the Andean range^27^. The range of *P. gillissi* has a Mediterranean climate, but also extended further south up to the Imperial River Basin where there is a higher influence of a temperate rainy climate^68^ (Fig. 6c). The predominant vegetation in the southern part of the range of *P. gillissi* includes deciduous forests^67^.

### Species’ occurrence data

A total of 285 georeferenced presence records were collected from multiple sources and included 107 records for *P. irwini* and 178 records for *P. gillissi*. These occurrences were obtained after 1950 and filtered using ArcGIS Pro 3.0.0 with license provided by Oregon State University (https://www.esri.com/en-us/arcgis/products/arcgis-pro/overview). All georeferenced records were obtained through the database of the Ministry of the Environment of the Chilean Government, Global Biodiversity Facility (GBIF) https://www.gbif.org/ in: 10.15468/dl.3bbucr, 10.15468/dl.g7v3pt and published literature (See Supplementary materials). Since the data from GBIF and the Chilean Government are freely accessible and no new samples were collected by the authors, ethical approval for the use of data was not required.

### Hydrological and geomorphological characterization

A synthetic network of hydrological features was generated to represent the spatial distribution of freshwater environments throughout the study area and characterized based on geomorphic (relatively constant over time) and downscaled climatic data (variable over time). Based on a Digital Elevation Model (DEM) at 30 m resolution from the Shuttle Radar Topography Mission (SRTM), we delineated all river basins of interest (n = 9) within our study region. Using flow accumulation algorithms (see details in Olivos *et al*^69^ and Jan *et al*^11^), we selected all drainage lines draining above 0.1 km^2^ and vectorized a digital stream network segmented every 100 m (hereafter stream reaches). We characterized stream reaches based on persistent geophysical attributes, namely drainage area (km^2^), average channel gradient (%), sinuosity (index), aspect (°), stream order (Strahler), valley confinement (index), and distance from each reach to the ocean (distance downstream, km^2^) and to the headwaters (distance upstream, km^2^). Additional attributes related to bioclimate (see section below) were added to each stream reach and climate change scenario.

### Bioclimatic variables and downscaling climate change scenarios

To obtain high spatial resolution of bioclimatic variables we conducted a statistical downscaling process of monthly climatic variables (minimum and maximum temperature and precipitation) from 1 km to 15 m resolution. This downscaling procedure is more appropriate to assess the impacts of climate change on natural habitats and biota^70^. The downscaling process was performed using the multivariate geographically weighted regression (GWR) method^71^. This method is useful to develop spatiotemporal regressions affected by parametric instability, providing adequate results to generate maps adjusted to different scales in cases of variables that vary spatially^71^. Specifically, we used Worldclim v2.1^72^ with a resolution of 1 km as the response variable, which was locally corrected by the GWR method using a series of meteorological data from the Dirección de Aguas de Chile (DGA) (See Supplementary material), and Pedreros *et al*^73^. As predictor, we used the digital elevation model DEM (SRTM of 30 m resolution resampled at a resolution of 1 km and 15 m. The spatial coefficients obtained through the GWR were interpolated using ORDINARY KRIGING with automatic variogram. All statistical analyzes were performed using the ‘spgwr’ and ‘hydroGOF’ packages^74,75^. More details on the downscaling methodology and GWR are provided in Fotheringham *et al*^71^ and Contador *et al*^76^. Based on these downscaled monthly climatic datasets (i.e., minimum temperature, maximum temperature, and precipitation), we obtained bioclimatic variables commonly used in SDMs using the 'biovars' function implemented in the ‘dismo’^77^ package for R statistical software^78^. We generated the 19 bioclimatic variables commonly invoked in SDMs. We re-calculated all precipitation-associated variables (bio12–bio19), replacing local precipitation values with averaged catchment precipitation. This resulted in eight additional bioclimatic variables (herein, “hydroclimatic” variables) representing monthly variability in discharge and hydrological conditions. See the Supplementary materials for the complete list of bioclimatic variables.

### Habitat model development

We used MaxEnt version 3.4.4^79^ for fitting our final habitat models for both species. We transferred MaxEnt models from a significant portion of the native ranges of both species to predict potentially suitable streams over their entire range using present and future climatic scenarios by 2050 and 2070 (SSP245 and SSP585). We used the ‘Kuenm’ R package^80^ to fit MaxEnt models using climatic and spatially continuous topographic variables (See Supplementary materials). This R package allowed for comparisons in MaxEnt among candidate models under different regularization multipliers, and feature classes, balancing predictive power with appropriate complexity and statistical significance. Candidate models were evaluated for partial ROC, omission rate (E) and model complexity (AICc), to select the best model for each species (Supplementary materials Fig. S1 and Fig. S2).

### Model evaluation

We evaluated our final models using randomized data partitioning. We used 70% of the occurrences to train the models and the remaining 30% to evaluate them. We replicated this randomly to generate 10 replicates to account for variability in model perfomance. On average, the optimized MaxEnt models showed 0% omission rate for *P. irwini* and 7% omission rate for *P. gillissi* on validation data.

The use of the Receiver Operating Characteristic (ROC) analysis for model evaluation has been criticized for giving equal weight to omission and commission errors^81^. Therefore, we used partial ROC (pROC) developed for ENM evaluation^82^. Partial ROC uses AUC ratios (the partial AUC divided by random expectation), where a value of 1.0 represented model performance no better than random, whereas models with AUC ratios near or greater than 2.0 were considered good^81^. The *P*-values of pROC indicated whether the ratio of model AUC to the random AUC is statistically significant. The details of evaluation metrics for our final MaxEnt models for each species are provided in the Supplementary materials Fig. S1 and Fig. S2.

### Mapping suitable habitats from model outputs

Subsequently, threshold cutoffs for each species were selected using the minimum training presence threshold (0.06 for *P. irwini* and 0.04 for *P. gillissi*)*.* Thus, the suitable habitats for the species were of unsuitability *P* ≤ 0.06 and *P* ≤ 0.04, respectively. The maps of the modeled present and future distribution of habitats for *P. irwini* and *P. gillissi* (Figs. 1 and 2) were built based on the probabilities (related presence) and categorized as unsuitable, moderately suitable (minimum training presence threshold ≤ *P* < 0.5), and highly suitable (0.5 ≤ *P* < 1.0) habitats for each species. We created maps of magnitude of change in suitable habitats between present and future climate change scenarios (intermediate SSP245 and extreme SSP585 in 2070) for *P. irwini* and *P. gillissi* (Fig. 5) only using suitable (moderately and highly) habitats. We calculated the percentage of stable habitats (suitable habitat without changes in the future), new habitats (suitable habitats found in the future, but not in the present) and lost habitats (suitable habitats in the present that are no longer found in the future) based on the total number of suitable habitats pixels. Lastly, we summarized densities of present and future habitats for *P. irwini* and *P. gillissi* (Fig. 3) using the probabilities from the best supported MaxEnt model using the bandwidth Bw = 60 for *P. irwini* and Bw = 20 for *P. gillissi*.

### Supplementary Information

Supplementary Information 1.

Supplementary Information 2.

Supplementary Information 3.

Supplementary Information 4.

Supplementary Figures.

Supplementary Tables.

## Data Availability

The datasets generated during and/or analysed during the current study will be available in the DRYAD repository, after the manuscript is accepted [10.5061/dryad.tht76hf5m]. However, the high-resolution layers created in this study will be available upon reasonable request from the corresponding author.

## References

[1] Dudgeon, D. *et al.* Freshwater biodiversity: Importance, threats, status and conservation challenges. *Biol. Rev.* **81**(2), 163–182. 10.1017/S1464793105006950 (2006). 16336747 10.1017/S1464793105006950

[2] Heino, J., Virkkala, R. & Toivonen, H. Climate change and freshwater biodiversity: Detected patterns, future trends and adaptations in northern regions. *Biol. Rev.* **84**(1), 39–54. 10.1111/j.1469-185X.2008.00060.x (2009). 19032595 10.1111/j.1469-185X.2008.00060.x

[3] Comte, L., Buisson, L., Daufresne, M. & Grenouillet, G. Climate-induced changes in the distribution of freshwater fish: Observed and predicted trends. *Freshw. Biol.* **58**(4), 625–639. 10.1111/fwb.12081 (2013). 10.1111/fwb.12081

[4] Poff, N. L., Brinson, M. M. & Day, J. W. *Aquatic Ecosystems and Global Climate Change* Vol. 44, 1–36 (Pew Center on Global Climate Change, 2002).

[5] Hof, C., Araújo, M. B., Jetz, W. & Rahbek, C. Additive threats from pathogens, climate and land-use change for global amphibian diversity. *Nature* **480**, 516–519. 10.1038/nature10650 (2011). 22089134 10.1038/nature10650

[6] Rieman, B. *et al.* Anticipated climate warming effects on bull trout habitats and populations across the interior Columbia River basin. *Trans. Am. Fish. Soc.* **136**(6), 1552–1565. 10.1577/T07-028.1 (2007). 10.1577/T07-028.1

[7] Pound, K. L., Larson, C. A. & Passy, S. I. Current distributions and future climate-driven changes in diatoms, insects, and fish in US streams. *Glob. Ecol. Biogeogr.* **30**(1), 63–78. 10.1111/geb.13193 (2021). 10.1111/geb.13193

[8] Skelly, D. *et al.* Evolutionary responses to climate change. *Conserv. Biol.* **21**(5), 1353–1355. 10.1111/j.1523-1739.2007.00764.x (2007). 17883501 10.1111/j.1523-1739.2007.00764.x

[9] Pearson, R. G. & Dawson, T. P. Predicting the impacts of climate change on the distribution of species: Are bioclimate envelope models useful?. *Glob. Ecol. Biogeogr.* **12**(5), 361–371. 10.1046/j.1466-822X.2003.00042.x (2003). 10.1046/j.1466-822X.2003.00042.x

[10] Cutler, J. S., Olivos, J. A., Sidlauskas, B. & Arismendi, I. Evaluating the distribution of freshwater fish diversity using a multispecies habitat suitability model to assess impacts of proposed dam development in Gabon, Africa. *Conserv. Sci. Pract.* **2**(2), e151. 10.1111/csp2.151 (2020). 10.1111/csp2.151

[11] Jan, A. *et al.* Habitat overlap among native and introduced cold-water fishes in the Himalayas. *Sci. Rep.* **13**, 15033. 10.1038/s41598-023-41778-y (2023). 37699910 10.1038/s41598-023-41778-y PMC10497582

[12] Peterson, A. T. & Holt, R. D. Niche differentiation in Mexican birds: Using point occurrences to detect ecological innovation. *Ecol. Lett.* **6**(8), 774–782. 10.1046/j.1461-0248.2003.00502.x (2003). 10.1046/j.1461-0248.2003.00502.x

[13] Soberón, J. & Nakamura, M. Niches and distributional areas: Concepts, methods, and assumptions. *Proc. Natl. Acad. Sci. U. S. A.* **106**, 19644–19650. 10.1073/pnas.0901637106 (2009). 19805041 10.1073/pnas.0901637106 PMC2780935

[14] Leroy, B. Choosing presence-only species distribution models. *J. Biogeogr.* **50**, 247–250. 10.1111/jbi.14505 (2022). 10.1111/jbi.14505

[15] Guisan, A. & Thuiller, W. Predicting species distribution: Offering more than simple habitat models. *Ecol. Lett.* **8**(9), 993–1009. 10.1111/J.1461-0248.2005.00792.X (2005). 34517687 10.1111/J.1461-0248.2005.00792.X

[16] Elith, J. & Leathwick, J. R. Species distribution models: Ecological explanation and prediction across space and time. *Ann. Rev. Ecol. Evol. Syst.* **40**(1), 677–697. 10.1146/annurev.ecolsys.110308.120159 (2009). 10.1146/annurev.ecolsys.110308.120159

[17] Fierro, P. *et al.* Assessment of anthropogenic threats to Chilean Mediterranean freshwater ecosystems: Literature review and expert opinions. *Environ. Impact Assess. Rev.* **77**, 114–121. 10.1016/j.eiar.2019.02.010 (2019). 10.1016/j.eiar.2019.02.010

[18] Jarić, I., Lennox, R. J., Kalinkat, G., Cvijanović, G. & Radinger, J. Susceptibility of European freshwater fish to climate change: Species profiling based on life-history and environmental characteristics. *Glob. Change Biol.* **25**(2), 448–458. 10.1111/gcb.14518 (2019). 10.1111/gcb.14518 30417977

[19] Radinger, J. & García-Berthou, E. The role of connectivity in the interplay between climate change and the spread of alien fish in a large Mediterranean river. *Glob. Change Biol.* **26**(11), 6383–6398. 10.1111/gcb.15320 (2020). 10.1111/gcb.15320 32813898

[20] Figueroa, R. *et al.* Freshwater biodiversity and conservation in Mediterranean climate streams of Chile. *Hydrobiologia* **719**, 269–289. 10.1007/s10750-013-1685-4 (2013). 10.1007/s10750-013-1685-4

[21] Vila, I. & Habit, E. Current situation of the fish fauna in the Mediterranean region of Andean river systems in Chile. *FiSHMED Fish. Mediterranean Environ.* **2**, 19. 10.29094/FiSHMED.2015.002 (2015). 10.29094/FiSHMED.2015.002

[22] Garreaud, R. *et al.* The central Chile mega drought (2010–2018): A climate dynamics perspective. *Int. J. Climatol.* **40**(1), 421–439. 10.1002/joc.6219 (2020). 10.1002/joc.6219

[23] Pino, P. *et al.* Chile confronts its environmental health future after 25 years of accelerated growth. *Ann. Glob. Health* **81**(3), 354–367. 10.1016/j.aogh.2015.06.008 (2015). 26615070 10.1016/j.aogh.2015.06.008 PMC4663014

[24] Habit, E. *et al*. Biodiversidad de Ecosistemas de Agua Dulce. Mesa Biodiversidad-Comité Científico COP25; Ministerio de Ciencia, Tecnología, Conocimiento e Innovación, 64 pp (2019).

[25] Ministerio de Medio Ambiente. Inventario nacional de especies de Chile (2022). Recovered from: http://especies.mma.gob.cl/CNMWeb/Web/WebCiudadana/Default.aspx

[26] Alò, D., Pizarro, V. & Habit, E. Fish body size influenced by multiple drivers. *Ecography*10.1111/ecog.06865 (2023). 10.1111/ecog.06865

[27] Vila, I., Fuentes, L. & Contreras, M. Peces límnicos de Chile. *Boletín Museo Historia Natural Chile* **48**, 61–75 (1999). 10.54830/bmnhn.v48.1999.362

[28] Piedra, P. *et al.* Movement patterns of the native fish fauna of the San Pedro River (Valdivia River Basin, Chile). *Gayana* **76**, 59–70. 10.4067/S0717-65382012000100006 (2012). 10.4067/S0717-65382012000100006

[29] Arratia, G. Géneros de peces de aguas continentales de Chile. *Museo Nacional de Historia Natural* **34**, 1–108 (1981).

[30] Campos, *et al*. Categorías de Conservación de peces nativos de aguas continentales de Chile. Boletín del Museo Nacional de Historia Natural, Santiago de Chile, 47, 101–122 (1998).

[31] Habit, E., & Belk, M. C. Threatened fishes of the world: *Percilia irwini* (Eigenmann 1927) (Perciliidae) (2007). 10.1007/s10641-006-0014-4

[32] García, A., Sobenes, C., Link, O. & Habit, E. Bioenergetic models of the threatened darter *Percilia irwini*. *Mar. Freshw. Behav. Physiol.* **45**(1), 17–28. 10.1080/10236244.2012.668283 (2012). 10.1080/10236244.2012.668283

[33] Vivancos, A. *et al.* Hydrological connectivity drives longitudinal movement of endangered endemic Chilean darter *Percilia irwini* (Eigenmann, 1927). *J. Fish Biol.* **98**(1), 33–43. 10.1111/jfb.14554 (2021). 32964414 10.1111/jfb.14554

[34] Hof, C. Towards more integration of physiology, dispersal and land-use change to understand the responses of species to climate change. *J. Exp. Biol.* **224**, 238352. 10.1242/jeb.238352 (2021). 10.1242/jeb.238352 33627466

[35] Valenzuela-Aguayo, F., McCracken, G. R., Manosalva, A., Habit, E. & Ruzzante, D. E. Human-induced habitat fragmentation effects on connectivity, diversity, and population persistence of an endemic fish, *Percilia irwini*, in the Biobío River basin (Chile). *Evol. Appl.* **13**(4), 794–807. 10.1111/eva.12901 (2020). 32211068 10.1111/eva.12901 PMC7086057

[36] Manosalva, A. & Górski, K. Percilia irwini. The IUCN Red List of Threatened Species 2023: e.T16584A176561864 (2023). 10.2305/IUCN.UK.2023-1.RLTS.T16584A176561864.en. Accessed on 24 June 2024.

[37] Manosalva, A. & Górski, K. Percilia gillissi. The IUCN Red List of Threatened Species 2023: e.T16584A176561865 (2023). 10.2305/IUCN.UK.2023-1.RLTS.T16584A176561865.en. Accessed on 24 June 2024.

[38] Falvey, M. & Garreaud, R. D. Regional cooling in a warming world: Recent temperature trends in the southeast Pacific and along the west coast of subtropical South America (1979–2006). *J. Geophys. Res. Atmos.*10.1029/2008JD010519 (2009). 10.1029/2008JD010519

[39] Burger, F., Brock, B. & Montecinos, A. Seasonal and elevational contrasts in temperature trends in Central Chile between 1979 and 2015. *Glob. Planet. Change* **162**, 136–147. 10.1016/j.gloplacha.2018.01.005 (2018). 10.1016/j.gloplacha.2018.01.005

[40] Crowley, T. J. Causes of climate change over the past 1000 years. *Science* **289**(5477), 270–277. 10.1126/science.289.5477.270 (2000). 10894770 10.1126/science.289.5477.270

[41] Xenopoulos, M. *et al.* Scenarios of freshwater fish extinctions from climate change and water withdrawal. *Glob. Change Biol.* **11**(10), 1557–1564. 10.1111/j.1365-2486.2005.001008.x (2005). 10.1111/j.1365-2486.2005.001008.x

[42] Barbarossa, V. *et al.* Threats of global warming to the world’s freshwater fishes. *Nat. Commun.* **12**(1), 1701. 10.1038/s41467-021-21655-w (2021). 33723261 10.1038/s41467-021-21655-w PMC7960982

[43] Comisión Nacional de Medio Ambiente (CONAMA, CL). Estudio de la variabilidad climática en Chile para el siglo XXI 63. Informe Final. Santiago, Chile. CONAMA (2006).

[44] Habit, E., Dyer, B. & Vila, I. Current state of knowledge of freshwater fishes of Chile. *Gayana* **70**(1), 100–113. 10.4067/S0717-65382006000100016 (2006). 10.4067/S0717-65382006000100016

[45] Marr, S. *et al.* Freshwater fish introductions in mediterranean-climate regions: Are there commonalities in the conservation problem?. *Divers. Distrib.* **16**(4), 606–619. 10.1111/j.1472-4642.2010.00669.x (2010). 10.1111/j.1472-4642.2010.00669.x

[46] Brook, B. W., Sodhi, N. S. & Bradshaw, C. J. A. Synergies among extinction drivers under global change. *Trends Ecol. Evol.* **23**(8), 453–460. 10.1016/j.tree.2008.03.011 (2008). 18582986 10.1016/j.tree.2008.03.011

[47] Díaz, G. *et al.* The longest fragment drives fish beta diversity in fragmented river networks: Implications for river management and conservation. *Sci. Total Environ.* **766**, 144323. 10.1016/j.scitotenv.2020.144323 (2021). 33418255 10.1016/j.scitotenv.2020.144323

[48] De Chazal, J. & Rounsevell, M. D. Land-use and climate change within assessments of biodiversity change: A review. *Glob. Environ. Change* **19**(2), 306–315. 10.1016/j.gloenvcha.2008.09.007 (2009). 10.1016/j.gloenvcha.2008.09.007

[49] Travis, J. M. J. Climate change and habitat destruction: A deadly anthropogenic cocktail. *Proc. Roy. Soc. Lond. Ser. B Biol. Sci.* **270**(1514), 467–473. 10.1098/rspb.2002.2246 (2003). 10.1098/rspb.2002.2246 PMC1691268 12641900

[50] Mooney, H. A. & Cleland, E. E. The evolutionary impact of invasive species. *Proc. Natl. Acad. Sci.* **98**(10), 5446–5451. 10.1073/pnas.091093398 (2001). 11344292 10.1073/pnas.091093398 PMC33232

[51] Heller, N. E. & Zavaleta, E. S. Biodiversity management in the face of climate change: A review of 22 years of recommendations. *Biol. Conserv.* **142**(1), 14–32. 10.1016/j.biocon.2008.10.006 (2009). 10.1016/j.biocon.2008.10.006

[52] Alofs, K. M., Jackson, D. A. & Lester, N. P. Ontario freshwater fishes demonstrate differing range-boundary shifts in a warming climate. *Divers. Distrib.* **20**(2), 123–136. 10.1111/ddi.12130 (2014). 10.1111/ddi.12130

[53] Lenoir, J. & Svenning, J.-C. Climate-related range shifts—A global multidimensional synthesis and new research directions. *Ecography* **38**(1), 15–28. 10.1111/ecog.00967 (2015). 10.1111/ecog.00967

[54] Heggenes, J., Bagliniere, J. L. & Cunjak, R. A. Spatial niche variability for young Atlantic salmon (*Salmo salar*) and brown trout (*S. trutta*) in heterogeneous streams. *Ecol. Freshw. Fish.* **8**(1), 1–21. 10.1111/j.1600-0633.1999.tb00048.x (1999). 10.1111/j.1600-0633.1999.tb00048.x

[55] Kelley, J. L., Grierson, P. F., Collin, S. P. & Davies, P. M. Habitat disruption and the identification and management of functional trait changes. *Fish Fish.* **19**(4), 716–728. 10.1111/faf.12284 (2018). 10.1111/faf.12284

[56] Nogués-Bravo, D., Rodríguez, J., Hortal, J., Batra, P. & Araújo, M. B. Climate change, humans, and the extinction of the woolly mammoth. *PLoS Biol.* **6**(4), e79. 10.1371/journal.pbio.0060079 (2008). 18384234 10.1371/journal.pbio.0060079 PMC2276529

[57] Elsen, P. R., Monahan, W. B. & Merenlender, A. M. Topography and human pressure in mountain ranges alter expected species responses to climate change. *Nat. Commun.* **11**(1), 2020. 10.1038/s41467-020-15881-x (1974). 10.1038/s41467-020-15881-x PMC7181879 32332913

[58] Thomas, C. *et al.* Extinction risk from climate change. *Nature* **427**(6970), 145–148. 10.1038/nature02121 (2004). 14712274 10.1038/nature02121

[59] Markovic, D., Freyhof, J. & Wolter, C. Where are all the fish: Potential of biogeographical maps to project current and future distribution patterns of freshwater species. *PLoS One* **7**(7), e40530. 10.1371/journal.pone.0040530 (2012). 22792361 10.1371/journal.pone.0040530 PMC3391242

[60] Keppel, G. *et al.* Refugia: identifying and understanding safe havens for biodiversity under climate change. *Glob. Ecol. Biogeogr.* **21**(4), 393–404. 10.1111/j.1466-8238.2011.00686.x (2012). 10.1111/j.1466-8238.2011.00686.x

[61] Nadeau, C. P., Giacomazzo, A. & Urban, M. C. Cool microrefugia accumulate and conserve biodiversity under climate change. *Glob. Change Biol.* **28**(10), 3222–3235. 10.1111/gcb.16143 (2022). 10.1111/gcb.16143 35226784

[62] Fourcade, Y. Comparing species distributions modelled from occurrence data and from expert-based range maps. Implication for predicting range shifts with climate change. *Ecol. Inf.* **36**, 8–14. 10.1016/j.ecoinf.2016.09.00 (2016). 10.1016/j.ecoinf.2016.09.00

[63] Faurby, S. & Araújo, M. B. Anthropogenic range contractions bias species climate change forecasts. *Nat. Clim. Change* **8**(3), 252–256. 10.1038/s41558-018-0089-x (2018). 10.1038/s41558-018-0089-x

[64] Lenoir, J., Hattab, T. & Pierre, G. Climatic microrefugia under anthropogenic climate change: Implications for species redistribution. *Ecography* **40**(2), 253–266. 10.1111/ecog.02788 (2017). 10.1111/ecog.02788

[65] Lembrechts, J. J., Nijs, I. & Lenoir, J. Incorporating microclimate into species distribution models. *Ecography* **42**(7), 1267–1279. 10.1111/ecog.03947 (2019). 10.1111/ecog.03947

[66] Valdés-Pineda, R. *et al.* Water governance in Chile: Availability, management, and climate change. *J. Hydrol.* **519**, 2538–2567. 10.1016/j.jhydrol.2014.04.016 (2014). 10.1016/j.jhydrol.2014.04.016

[67] Smith-Ramírez, C., Armesto, J. J. & Valdovinos, C. *Historia, biodiversidad y ecología de los bosques costeros de Chile* 708 (Editorial Universitaria, 2005).

[68] Di Castri, F. & Hajek, E. R. *Bioclimatología de Chile* Vol. 128, 163 (Vicerrectoría Académica de la Universidad Católica de Chile, 1976).

[69] Olivos, J. *et al.* An environmental resistance model to inform the biogeography of aquatic invasions in complex stream networks. *J. Biogeogr.* **50**, 1422–1436. 10.1111/jbi.14621 (2023). 10.1111/jbi.14621

[70] Leihy, R. I., Duffy, G. A., Nortje, E. & Chown, S. L. High resolution temperature data for ecological research and management on the Southern Ocean Islands. *Sci. Data* **5**(1), 1–13. 10.1038/sdata.2018.177 (2018). 30179229 10.1038/sdata.2018.177 PMC6122169

[71] Fotheringham, A. S., Charlton, M. & Brunsdon, C. Two techniques for exploring non-stationarity in geographical data. *Geogr. Syst.* **4**(1), 59–82 (1997).

[72] Fick, S. E. & Hijmans, R. J. WorldClim 2: New 1-km spatial resolution climate surfaces for global land areas. *Int. J. Climatol.* **37**(12), 4302–4315. 10.1002/joc.5086 (2017). 10.1002/joc.5086

[73] Pedreros, P. *et al.* Comportamiento térmico en ríos mediterráneos andinos de la zona centro-sur de Chile. *Limnetica* **32**(1), 87–96. 10.1016/j.limno.2020.125763 (2013). 10.1016/j.limno.2020.125763

[74] Zambrano-Bigiarini, M. Package ‘hydroGOF’. Goodness-of-fit Functions for Comparison of Simulated and Observed (2017).

[75] Bivand, R., Yu, D., Nakaya, T., Garcia-Lopez, M. A., & Bivand, M. R. Package ‘spgwr’. R software package (2017).

[76] Contador, T. *et al.* Assessing distribution shifts and ecophysiological characteristics of the only Antarctic winged midge under climate change scenarios. *Sci. Rep.* **10**(1), 9087. 10.1038/s41598-020-65571-3 (2020). 32493944 10.1038/s41598-020-65571-3 PMC7270094

[77] Hijmans, R. J., Phillips, S. J., Leathwick, J., Elith, J., & Hijmans, M. R. Dismo: Species distribution modeling. R package version: 1-0 (2015).

[78] R Core Team, A., & R Core Team. R: A language and environment for statistical computing. R Foundation for Statistical Computing, Vienna, Austria. 2012 (2022).

[79] Phillips, S. J., Anderson, R. P., Dudík, M., Schapire, R. E. & Blair, M. E. Opening the black box: An open-source release of Maxent. *Ecography* **40**(7), 887–893. 10.1111/ecog.03049 (2017). 10.1111/ecog.03049

[80] Cobos, M. E., Peterson, A. T., Osorio-Olvera, L. & Jiménez-García, D. An exhaustive analysis of heuristic methods for variable selection in ecological niche modeling and species distribution modeling. *Ecol. Inf.* **53**, 100983. 10.1016/j.ecoinf.2019.100983 (2019). 10.1016/j.ecoinf.2019.100983

[81] Escobar, L. E., Qiao, H., Cabello, J. & Peterson, A. T. Ecological niche modeling re-examined: A case study with Darwin’s fox. *Ecol. Evol.* **8**(10), 4757–4770. 10.1002/ece3.4014 (2018). 29876055 10.1002/ece3.4014 PMC5980497

[82] Peterson, A. T., Papeş, M. & Soberón, J. Rethinking receiver operating characteristic analysis applications in ecological niche modeling. *Ecol. Model.* **213**(1), 63–72. 10.1016/j.ecolmodel.2007.11.008 (2008). 10.1016/j.ecolmodel.2007.11.008

