# Author Correction: Climate change can disproportionately reduce habitats of stream fishes with restricted ranges in southern South America

**DOI:** 10.1038/s41598-024-68912-8

**Published:** 2024-08-02

**Authors:** Gustavo Bizama, Arif Jan, J. Andrés Olivos, Guillermo Fuentes‑Jaque, Claudio Valdovinos, Roberto Urrutia, Ivan Arismendi

**Affiliations:** 1 Doctorado de Ciencias Ambientales, en Ecosistemas Acuáticos Continentales, Facultad de Ciencias Ambientales, Centro EULA‑Chile, Universidad de Concepción, Víctor Lamas 1290, 4070386 Concepción, Chile; 2 Centro de Recursos Hídricos Para La Agricultura y Minería CRIHAM, Concepción, Chile; 3 Department of Fisheries, Wildlife, and Conservation Sciences, Oregon State University, Corvallis, OR 97331 USA; 4 Department of Environmental Sciences and Renewable Natural Resources, Faculty of Agricultural Sciences, University of Chile, Santiago, Chile

Correction to: *Scientific Reports* 10.1038/s41598-024-66374-6, published online 09 July 2024

The original version of this Article contained an error in the Acknowledgements where a project number was incorrect.

“GB was funded by the National Agency for Research and Development of Chilean Government ANID, National Doctorate Scholarship 2020, Grant N° 21201511, and RU thanks Project ANID/FONDAP/15130015.”

now reads:

“GB was funded by the National Agency for Research and Development of Chilean Government ANID, National Doctorate Scholarship 2020, Grant N° 21201511, and RU thanks Project ANID/FONDAP/1523A0001.”

The original Article has been corrected.

