# Supplementary material for: Climate change can disproportionately reduce habitats of stream fishes with restricted ranges in southern South America
Source: Sci Rep. 2024 Jul 9;14:15780. doi: 10.1038/s41598-024-66374-6 (PMC11238036; doi:10.1038/s41598-024-66374-6)
Supplement: Supplementary file 5 — Supplementary Figures. [file 41598_2024_66374_MOESM5_ESM.docx]

**Supplementary information**

Model selection for *P. irwini*


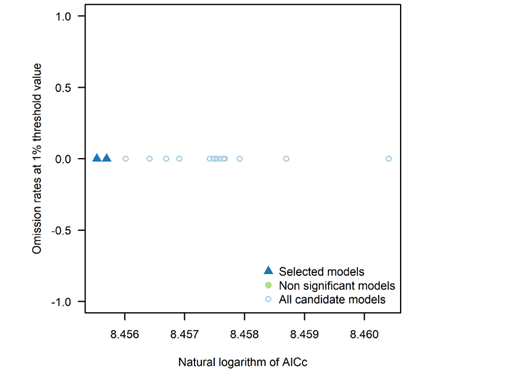


**FIG.S1.** Model selection for *P. irwini*

Model selection for *P. gillissi*


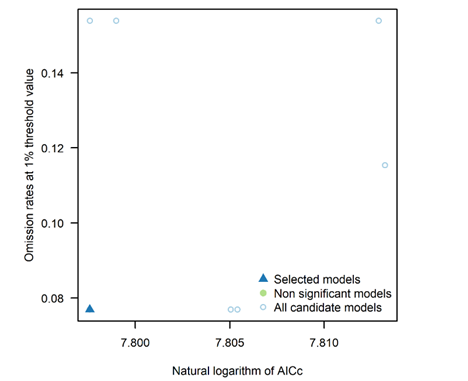


**FIG.S2.** Model selection for *P. gillissi.*

**Table S2** Performance statistics for models selected based on the users pre-defined criteria.

| **Species** | **Omission rate** | **Mean AUC ratio** | **Partial ROC** | **W_AICc** | **AICc** | **N parameters** |
| --- | --- | --- | --- | --- | --- | --- |
| *P. irwini* | 0.00 | 1.993 | 0.00 | 0.437 | 4701.016 | 11 |
| *P. gillissi* | 0.07 | 9.624 | 0.00 | 1 2434.73 14 | | |
