# Supplementary material for: Climate change can disproportionately reduce habitats of stream fishes with restricted ranges in southern South America
Source: Sci Rep. 2024 Jul 9;14:15780. doi: 10.1038/s41598-024-66374-6 (PMC11238036; doi:10.1038/s41598-024-66374-6)
Supplement: Supplementary file 6 — Supplementary Tables. [file 41598_2024_66374_MOESM6_ESM.docx]

| Variable | Description |
| --- | --- |
| \| **Strahler** \| \| --- \| | Stream order according to Strahler |
| Bio 11 | Mean Temperature of Coldest Quarter |
| Bio 1 | Annual Mean Temperature |
| Bio 10 | Mean Temperature of Warmest Quarter |
| Bio 4 | Temperature Seasonality (standard deviation ×100) |
| Bio 15 hydro | Precipitation Seasonality (Coefficient of Variation) (catchment) |
| Bio 12 hydro | Average Annual Precipitation (catchment) |
| vwi^1^ | Valley-to-channel width ratio (channel confinement) where lines running through the narrowest canyons have an index of 1, and a lines running through wide alluvial valleys have value of 100 |
| average slope | Average channel slope or gradient in percent of elevation change |

Table S1. Variables selected for the SDMs of *P. irwini* and *P. gillissi*. ^1^ Olivos, J. A., Arismendi, I., Penaluna, B. E., Flitcroft, R., Huertas Herrera, A., Firman, J., & Giannico, G. (2023). An environmental resistance model to inform the biogeography of aquatic invasions in complex stream networks. Journal of Biogeography, 50, 1422–1436. <https://doi.org/10.1111/jbi.14621>
